# Gastrointestinal Stromal Tumor (GIST) and Synchronous Intra-Abdominal Liposarcoma: A Report of Two Rare Cases and Literature Review

**DOI:** 10.1155/2021/2626635

**Published:** 2021-09-01

**Authors:** Alexandros Diamantis, Athina A. Samara, Ioannis Baloyiannis, Dimitrios Symeonidis, Andreas-Marios Diamantis, Maria Tolia, Konstantinos Bouliaris, Georgios Koukoulis, Konstantinos Tepetes

**Affiliations:** ^1^Department of General Surgery, University Hospital of Larissa, Larissa, Greece; ^2^Department of Radiotherapy, University of Crete, Heraklion, Greece; ^3^Department of General Surgery, General Hospital of Larissa, Larissa, Greece; ^4^Department of Pathology, University Hospital of Larissa, Larissa, Greece

## Abstract

**Introduction:**

Gastrointestinal stromal tumors (GISTs) quite frequently occur synchronously with other malignancies, with most cases being adenocarcinomas. GISTs and liposarcomas are both of mesenchymal origin, and their coexistence is extremely rare.

**Methods:**

We conducted a review of the current literature regarding the synchronous occurrence of GISTs and intra-abdominal liposarcomas. An electronic search of the literature was undertaken using MEDLINE (database provider PubMed). Furthermore, we present the first described case of an 86-year-old male with a GIST and synchronous liposarcoma, both located in the stomach, as well as a 66-year-old male with a gastric GIST and concurrent retroperitoneal liposarcoma.

**Results:**

A total of 5 cases of synchronous GIST and intra-abdominal liposarcoma have been reported in the literature to date, with the most recent cases included in the present study.

**Conclusion:**

Further research is required to explain any possible correlation in the coexistence of these different neoplasms of the same origin. Meanwhile, R0 resection of both tumors remains the treatment of choice.

## 1. Introduction

Although rare and accounting for only 0.1–1% of all malignancies of the gastrointestinal (GI) tract, gastrointestinal stromal tumors (GISTs) are the most common nonepithelial neoplasms of the GI tract [1–6]. They are typically located in the stomach (50–60%), followed by the small intestine (30–40%), the colon-rectum (5–10%), and rarely the oesophagus (<5%). Less frequently, GISTs may develop in extra-GI sites, primarily affecting the mesentery, the omentum, and the retroperitoneum [2–6].

Conversely, liposarcomas are one of the most common soft-tissue sarcomas, usually affecting the limbs, retroperitoneum, and trunk [7]. Gastric liposarcomas are extremely rare, and mere 39 cases have been reported worldwide, with the antrum being the main location [8, 9]. Although GISTs quite often (in up to 38% of cases) coexist with other primary tumors [10], only three cases of synchronous GIST and intra-abdominal liposarcoma have been reported in the literature [11–13].

Herein, we present, for the first time in the literature to date, a patient with a GIST and synchronous liposarcoma both located in the stomach and a second case of a patient with a gastric GIST and a synchronous retrocecal liposarcoma. Furthermore, a review of the literature was conducted aiming to identify all cases of concurrent GIST and liposarcoma which have been reported to date.

## 2. Case Reports

### 2.1. Case A

**Figure 1:**
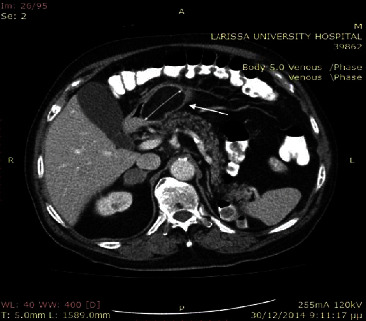
The computed tomography revealed a 5.6 cm × 3.5 cm hypodense soft-tissue mass in the antrum of the stomach (white arrows).

An 86-year-old male patient presented to the emergency department with a two-week history of fatigue and tarry stools. There were no significant findings based on physical examination. His past medical history included diabetes mellitus, hypertension, and chronic usage of acetylsalicylic acid. From routine laboratory exams, the patient's hematocrit was extremely low (HCT = 19%), while all the other parameters were within normal limits. An emergency upper gastrointestinal endoscopy was performed and revealed a submucous tumor in the gastric antrum, without present bleeding. The patient received a blood transfusion and intravenous iron therapy and remained hemodynamically stable. A computed tomography (CT) examination was performed which revealed a hypodense and well-defined soft-tissue mass (5.5 × 3.5 cm) in the antrum of the stomach (Figure 1). No hepatic metastasis or nodal involvement was detected.

Two tumors were identified intraoperatively. The larger tumor was detected in the antrum and the smaller tumor in the gastric body. No distant metastasis or nodal involvement was noted. Distal gastrectomy, Billroth II reconstruction, and Braun enteroenterostomy were performed. The patient had an uneventful recovery and was discharged on the 9^th^ postoperative day.

**Figure 2:**
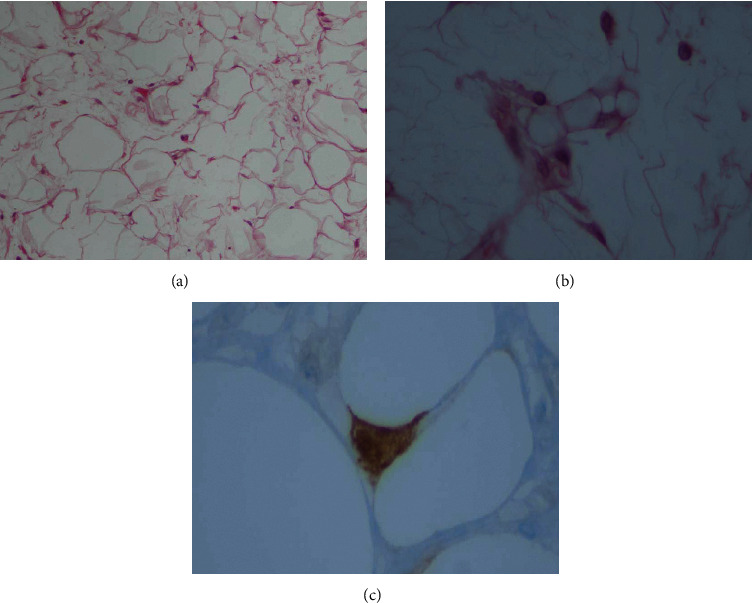
(a) An adipocytic tumor was observed. The tumor cells showed appreciable anisocytosis and focal anisonucleosis without obvious pleomorphism. H + E, initial magnification 100x. (b) There were rare, sporadic cells with the morphologic appearance of a lipoblast. H + E, initial magnification 400x. (c) Several of the atypical adipocytes and lipoblasts showed immunoreactivity with p16 on immunohistochemistry, initial magnification 400x.

In histopathological examination, the gastric body tumor (*d* = 11 mm, R0 resection) was a low-risk GIST (1-2 mitoses/50 hpf). Immunohistochemistry displayed strong staining for c-Kit, CD34, and DOG1. The second tumor in the antrum (*d* = 55 mm, R0) was a well-differentiated liposarcoma (Figure 2), positive for p16 immunohistochemistry. None of the lymph nodes excised were infiltrated.

The patient did not undergo any adjuvant treatment, and in his follow-up 5 years after surgery, the patient remains disease-free. Due to the patient's age, future follow-up was terminated.

### 2.2. Case B

A 66-year-old male patient visited the emergency department in a hemodynamically unstable state with bloody vomiting. Based on his past medical history, the patient reported diabetes mellitus, hypertension, and smoking. An emergency upper gastrointestinal endoscopy was performed, which revealed a submucous tumor in the wall of the gastric antrum with present acute hemorrhage. The bleeding could not be controlled endoscopically, and a decision was taken to perform an urgent surgical operation.

During the exploratory laparotomy, a medium-sized tumor behind the cecum was also detected. A distal gastrectomy with Billroth II reconstruction and resection of the retrocecal mass were performed. The postoperative course was uneventful, and the patient was discharged on the 7^th^ postoperative day.

Histopathological examination showed a mesenchymal gastric stromal tumor (GIST) with a diameter of 4 cm. The risk of progressive disease was low (4–6/50 hpf), and immunohistochemistry displayed strong staining for c-Kit, CD34, and S-100 protein. Histopathological findings for the retrocecal tumor revealed a 5.9 cm well-differentiated sclerosing-type liposarcoma with immunohistochemistry S-100 protein and vimentin positive. Cancer cells were present microscopically at the resection margin (R1 resection) of the liposarcoma. Thus, adjuvant chemotherapy (palbociclib) was introduced.

At the 6-month follow-up, the patient remained disease-free; however, unfortunately, 13 months after surgery, the patient suffered a severe myocardial infarction and died 2 days later in the intensive care unit.

## 3. Summary

The main clinical and pathological features of all 5 patients with two synchronous mesenchymal tumors are summarized in Table 1. The mean age of patients involved was 618 years ranging from 46 to 86 years of age and affecting more frequently the male gender (60%). In all but one case, the GISTs were small (mean size: 3.02 cm) affecting more frequently the stomach (80%), with no or low mitotic rate and classified as very low risk for malignant potential. The liposarcomas ranged from 3 cm to 25 cm, affecting the retroperitoneum in three patients, the mesentery in two patients, and the stomach in only one patient. In 3 cases, the grade was G1, and in one case, the grade was G2, while in the case described by Zhou et al., information about the liposarcomas' grades was not provided. Three of the patients received adjuvant therapy as shown in Table 1. The follow-up period described in the above cases varied from 2 months to 72 months. None of the patients had a known relapse of the disease, and only one patient died from heart infarction 13 months after surgery [9–11].

## 4. Discussion

Although gastrointestinal stromal tumors (GISTs) are rare neoplasms, they represent the most common mesenchymal tumors of the gastrointestinal tract. GISTs originate from the cells of Cajal, which are responsible for autonomous gastrointestinal (GI) movement [4–6, 14, 15]. There is a peak in the incidence of GISTs in adults between 55 and 65 years of age [4, 15]. While the majority occur as sporadic solitary lesions, they may also develop in a familial fashion, such as in neurofibromatosis and Carney triad [16]. Fletcher et al. proposed the risk classification of aggressive behavior in GISTs [17]. They are classified into four risk groups (very low, low, intermediate, and high risk) based on tumor size and mitotic count [17].

Liposarcoma is the most common soft-tissue sarcoma, accounting for 15%–20% of all mesenchymal malignancies. Based on morphological and histopathological features, they are classified into 4 types: (1) well differentiated; (2) dedifferentiated; (3) myxoid/round cell; and (4) pleomorphic [18]. Well-differentiated liposarcomas have a high rate of local recurrence, but minimal metastatic potential and 5-year survival can reach 90%. Dedifferentiated lesions have similar local effects, and although they can metastasize systemically, their 5-year survival can reach 75%. In addition, patients with low-grade myxoid liposarcomas, defined as <5% round cell areas, have a 5-year survival of 90%. Patients with high-grade myxoid/round cell liposarcoma (>5% round cells) have a 5-year survival probability of 60%. Pleomorphic liposarcomas, the rarest subtype, have the poorest prognosis with a high metastatic potential and a 5-year survival probability of 30–50% [18].

GISTs commonly coexist with other primary tumors that can involve either the GI tract or other extra-GI sites. According to a recently published review, the synchronous occurrence of GISTs with other malignancies varies from 8% to 38%, while the most frequent localization of GIST-associated malignancies is in the gastrointestinal tract. Interestingly, GISTs of small size and low risk of malignant transformation are usually found in patients with other malignancies, supporting the theory that the GIST itself in these patients has little effect on the overall survival and patients' prognosis [8, 19]. It is not yet clear if a causal association exists or if the concomitant occurrence of GISTs with other malignancies is coincidental. Such an occurrence has mainly been described in the literature through case reports and rarely through case series; this literature is insufficient to prove if an association between these two entities exists [20, 21].

As yet, the clinical importance of this concurrence has not been clarified. In a recent case series study of survival analysis comparing the overall survival, no statistically significant difference was found between patients with a single or a synchronous GIST. However, patients with synchronous GISTs and other primary tumors had a statistically significant increased possibility for relapse. In addition, they proved that patients with a single GIST have larger tumors than patients with synchronous tumors, and the difference in the mean age of appearance was not statistically significant in the two groups [22].

Both GISTs and liposarcomas are of mesenchymal origin, and their coincidence requires specific expertise related to diagnostic and therapeutic management [23]. Possible misinterpretation of the smaller mass as a metastasis poses an additional challenge for the surgeon, leading to a potential false treatment choice [8]. Radical surgery is the gold-standard treatment for all mesenchymal tumors on the grounds that R0 resection remains the most significant prognostic factor. Radiotherapy and chemotherapy show only a limited efficiency in these cases. Conversely, since the introduction of tyrosine kinase inhibitors such as imatinib and sunitinib, patients with intermediate- and high-risk GISTs have an additional targeted therapy choice in an adjuvant or neoadjuvant setting, ameliorating the overall and disease-specific survival [23]. However, as previously stated, GISTs are usually of small size and low risk in patients with synchronous malignancies, and subsequently, in most cases, they do not require any other adjuvant treatment [8].

We identified only three previously reported cases in the literature. The first case reported by Diamantis et al. was a 46-year-old patient with synchronous gastric GIST and retroperitoneal liposarcoma, treated with excision and adjuvant chemoradiotherapy due to microscopically positive margins in the liposarcoma specimen [10]. The second case was a coincident carcinoid tumor of the abdomen, a GIST, and a mesentery liposarcoma in a 55-year-old female NF1 patient, treated only with surgical resection [11]. Finally, the third case reported by Sonoda et al. was a 56-year-old patient with synchronous gastric GIST and two liposarcomas, one in the mesentery and the other in the retroperitoneum, treated with resection and adjuvant radiotherapy to the former liposarcoma bed [9].

## 5. Conclusion

The synchronous occurrence of GIST and liposarcoma is very rare, with only three cases described in the literature worldwide. Here, we report two additional cases, one of which is the first case of synchronous GIST and liposarcoma both located in the stomach. Although coincidence may be the answer, the hypothesis of gene mutations or a carcinogenic agent resulting in two tumors of the same origin cannot be excluded. Nevertheless, further research is required to explain the possible causation of the synchronous occurrence of two mesenchymal tumors and define the best treatment strategy. Until then, the treatment of choice is radical excision of both tumors.

## Figures and Tables

**Table 1:** Main clinical characteristics and histopathological features.

Author (year)	Gender	Age	Location		Size (cm)		Risk stratification/grade		Adjuvant treatment	Follow-up (months)
			GIST	Liposarcoma	GIST	Liposarcoma	GIST	Liposarcoma		
Kang et al. (2013) [8]	F	46	Stomach	Retroperitoneum	6.5	3	High	G2	Radiochemotherapy	Disease-free (72 months)

Sonoda et al. (2014) [9]	F	55	Jejunum	Mesentery	1	3.5	None	G1	No	Disease-free (2 months)

Tepetes et al. (2018) [7]	M	56	Stomach	Retroperitoneum and mesentery	2.5	5 and 25	Very low	NA	Radiotherapy	Disease-free (15 months)

Diamantis et al. (2020)	M	86	Stomach	Stomach	1.1	5.5	None	G1	No	Disease-free (60 months)

Diamantis et al. (2021)	M	66	Stomach	Retroperitoneum	4	5.9	Very low	G1	Chemotherapy	Deceased (13 months)

NA: nonavailable.

## Data Availability

The data used to support the findings of this study are available from the corresponding author upon reasonable request.
